# Low Levels of Awareness of Lead Hazards among Pregnant Women in a High Risk—Johannesburg Neighbourhood

**DOI:** 10.3390/ijerph121214968

**Published:** 2015-11-27

**Authors:** Tanya Haman, Angela Mathee, Andre Swart

**Affiliations:** 1 Environment & Health Research Unit, South African Medical Research Council, P.O. Box 87373, Houghton 2041, Johannesburg, South Africa; Angie.Mathee@mrc.ac.za; 2 Faculty of Health Sciences, University of Johannesburg, P.O. Box 524, Auckland Park 2006, Johannesburg, South Africa; andres@uj.ac.za

**Keywords:** pregnant women, lead exposure, children, low awareness

## Abstract

Background: The widespread use of lead and elevated risk of lead exposure in South African children justifies a need for high levels of awareness of the sources, exposure pathways, and measures to reduce this risk in children. This study aimed to determine the levels of knowledge of lead hazards among pregnant women in an area where children had already been established to be at a high risk of lead exposure and poisoning. Methods: Following informed consent, a structured questionnaire was administered to 119 pregnant women attending antenatal clinic services at Rahima Moosa Mother and Child Hospital, west of central Johannesburg. Questions were asked about social, demographic and residential characteristics, as well as knowledge, perceptions, behaviours and practices in relation to child lead hazards. Conclusion: Overall awareness of the dangers of lead in pregnancy was low (11%). Amongst those who had heard of it, only 15% thought that lead could cause detrimental health effects. A consequence of this low level of awareness of lead hazards is a high potential for the participants and their children to unwittingly be exposed to environmental lead from various sources, thereby undermining preventative approaches.

## 1. Introduction

Lead is a toxic metal that is used in a wide range of products on a daily basis. Its widespread use has caused lead to be a global environmental contaminant, especially in urban areas [1]. Lead from various sources may concentrate in dust and soil and through the course of normal play, children may ingest lead-contaminated dust or soil, resulting in elevated blood lead burdens. Due to higher ingestion, respiration and metabolic rates, incompletely developed organ systems, and their natural tendency for exploratory behaviour, children are particularly vulnerable to lead exposure [2,3]. Children with pica for items such as soil and paint tend to have particularly high blood lead levels [4].

### 1.1. Health Effects of Lead

Among the health effects associated with elevated blood and bone lead levels are reductions in IQ scores, hyperactivity, shortened concentration spans, poor performance at school, anaemia, violent or aggressive behavior, and lowered lifetime achievements and earnings [5,6]. Many of the health effects of lead are irreversible [1,7]. A study conducted by Jedrychowski *et al.* (2009) showed similarities with earlier studies that found an association between elevated foetal lead exposure and low birth weight, abnormal development of organs, and later socio-behavioural developmental effects [3]. In addition, Naicker *et al*. (2011) reported significant associations between early lead exposure and socio-behavioural effects in teenaged participants in the Birth to Twenty Cohort study in South Africa [8]. Elevated lead exposure is a particular concern during pregnancy, since lead may cross the placental barrier, leading to foetal lead exposure [9]. Unborn and young children are especially vulnerable to the neurological effects of lead as the developing nervous system absorbs a higher fraction of lead [10].

Several studies undertaken in African settings point to a wide range of ongoing, contemporary sources of lead exposure in African communities [11]. For example, a recent study pointed to a high prevalence of geophagia (the ingestion of soil, clay or stones) at an antenatal clinic in Johannesburg [12,13]. Around one-fifth of pregnant women were reported to be geophagic [14]. Studies in New York have shown elevated lead exposure in pregnant women with geophagia [15]. Evidence of lead exposure has also been found in children’s playgrounds [16], mining towns [17], subsistence fishing villages [18], and in traditional medicines [19]. Recent incidents of large-scale, community-wide lead poisoning occurred in Zamfara, Nigeria, from informal gold mining [20] and in Dakar, Senegal, from dismantling batteries [21], emphasising the ongoing risk of lead poisoning in African communities.

### 1.2. Lead Hazard Awareness in Pregnant Women

Given the widespread use and elevated risk of lead exposure in African communities, it is important that caregivers be aware of the sources, pathways of exposure and measures to reduce the potential risks in their children. Low levels of awareness of lead hazards present an obstacle to efforts aiming to prevent lead poisoning in children, while high levels of awareness have proven to be instrumental in reducing childhood lead exposure [22]. Lead hazard awareness programmes are thus a vital foundation for effective lead exposure reduction measures [23] in Africa. Mothers, as primary caregivers, play an instrumental role in determining and managing the exposure of children to lead hazards. This study was conducted to determine the levels of knowledge of lead hazards in a group of pregnant women attending antenatal clinics in a neighbourhood of Johannesburg, where a previous blood lead survey of grade 1 children determined a high risk of lead exposure [24].

## 2. Methods

Following written informed consent, a structured questionnaire was administered to 148 pregnant women attending antenatal clinic services at the Rahima Moosa Mother and Child Hospital, situated to the West of central Johannesburg in the Gauteng Province of South Africa. A total of 29 eligible women declined to participate, mainly because of a lack of interest in the study topic. Ethical approval for the study was granted by the Ethics Committee of the University of the Witwatersrand, South Africa (M050334), and the project was approved by the Gauteng Provincial Department of Health.

One hundred and nineteen participants provided enough statistical power based on an estimated moderate effect size (0.5) and alpha (α = 0.05) between high- and low-risk behaviours. In addition, this sample size was thought to provide enough variability in responses to inform the intervention that is specific to a population of pregnant women with a similar cultural and socio-demographic profile. Convenience sampling was used to select pregnant women who attended the routine antenatal clinics at Rahima Moosa Mother and Child Hospital. Any pregnant woman attending routine antenatal clinics, older than 18 years of age, was asked to participate in the study.

A structured questionnaire was uniformly administered to all of the participants by the interviewers. Information was collected on the knowledge, perceptions and behaviours of pregnant women in relation to child lead hazards. Interviews were conducted in a reasonably quiet location to ensure privacy, in close proximity to the queue so that participants retained their positions in the queue. The data collection tool was an exploratory, descriptive and structured questionnaire which consisted of open- and closed-ended questions that were divided into the following sections: (a) socio-demographic characteristics and attributes; (b) information of the home environment; and (c) knowledge and perceptions, behaviours, cleaning practices and smoking of the participants in relation to the study objectives. Questionnaires were translated in English (90%), Afrikaans (3%), Sotho (5%) and Zulu (2%).

## 3. Results

The average age of the participants was 27 years (see Table 1). Their average household size was four persons (range: 1 to 17). The majority of the participants lived in free-standing dwellings, such as apartments and houses. In 94% of dwellings, electricity was the main fuel source used for daily cooking. In 7% of households, a member worked in the building or renovation domain, while 7% and 6%, respectively, worked in motor vehicle repairs (including spray painting) and electrical repairs. In 3%, someone worked with guns and ammunition, while in a further 3% of households, members worked with lead solder. Lead was used or lead-related activities were being undertaken in the homes of at least 18% (*n* = 22) of participants, including for fixing electrical appliances, spray painting of motor vehicles and recycling of scrap metal. Eighteen percent of study participants described their homes as “very dusty”. The study results indicated the potential for the participants and their children to be exposed to other sources of lead in the home environment, such as peeling lead-based paint, and living in old and dilapidated housing.

**Table 1:** Profile of study population (*n* = 119).

Characteristic	*N* (%)
Mean age, range	27 (18–43)
Education level	
No schooling and primary	14 (12)
Secondary	77 (65)
Tertiary	27 (23)
History of smoking during pregnancy	12 (10)
Exposure to environmental tobacco smoke	31 (26)
Living in free-standing dwelling or apartment	95 (80)
Perception of household dust	
Very dusty	21 (18)
Slightly dusty	64 (54)
Not dusty at all	33 (28)
Living in a house with indoor peeling paint	24 (20)
Living in a house with external peeling paint	26 (22)
Living in a house with bare soil backyard	25 (21)
Living in a house >25 years	46 (39)

Overall, mothers had a low level of awareness of lead. Only 11% had heard of lead before, mostly in relation to leaded petrol. There appeared to be little or no knowledge of other domestic sources of exposure to lead. Amongst those who had heard of lead, only 15% thought that lead could cause detrimental health effects in humans.

While most participants reported cleaning their floors on a daily basis, the majority used a dry broom. Similarly, most respondents (70%) said they dusted their homes daily and reported using a dry cloth for dusting. Sixty-seven percent (*n* = 112) of participants planned to breast feed, 6% to bottle feed and the remaining 27% to use a combination of these methods to feed their babies. Of the 101 participants who planned exclusive or some use of bottle feeding, 92% indicated that they would prepare their baby’s bottle-feeds with water boiled in a kettle or a pot, whereas 3% intended to use hot water from a tap and the remaining 5% did not know how they would prepare the bottle-feeds.

## 4. Discussion

This study has shown that despite the overwhelming presence of lead contamination, levels of knowledge of lead hazards are low, even in communities who have previously been established to be at risk of lead exposure [25]. Only 11% of the study participants had heard of lead before, mostly relating it to lead in petrol. Lead hazard awareness programmes, targeted especially at parents and pregnant women, have the potential to address this significant public health knowledge gap, and reduce the risk of lead poisoning in young and unborn children. Such awareness programmes should cover locally relevant sources of lead, for example paint, lead used in cottage industries, para-occupational lead exposure and hobbies involving lead. Awareness campaigns aimed at preventing lead exposure should also focus on high-risk practices and behaviours, such as excessive mouthing and pica, nutrition, tobacco smoking, the preparation of infant formula feeds with hot tap water [26], and housekeeping methods in South Africa [27]. Improved house cleaning practices proven to be a low-cost intervention for lead poisoning have shown potential for lowering environmental lead levels in Johannesburg dwellings [16] and elsewhere. For example, damp sweeping or mopping, as well as damp dusting, rather than using dry brooms or dusters [28] have been shown to lower lead concentrations in household dust, especially when used in combination with detergents [29]. In a society such as South Africa, where inequality, poverty and housing degradation is widespread, where multiple sources of lead prevail, and where the costs of environmental rehabilitation are exorbitant, it is vital that low-cost lead exposure reduction measures, such as public education and other awareness drives that will improve fundamental knowledge about lead hazards and its associated effects, be prioritized and scaled up.

## 5. Conclusions

While some of the interventions required to reduce the risk of childhood lead exposure may be costly and challenging (such as removal of old lead-based paint), the cost of lead hazard awareness programmes is relatively low, and they have been demonstrated to be effective. Despite some effort made to increase public awareness of lead hazards in South Africa, these have been neither widespread nor sustained. In a country such as South Africa, where many health challenges compete for limited resources, it is imperative that a goal of high levels of public awareness of lead hazards becomes a cornerstone of a national lead-poisoning prevention strategy.
